# Concerning Dental Literature and Dentistry in General, and Dr. Allen a Little

**Published:** 1861-12

**Authors:** Wm. A. Pease


### CONCERNING DENTAL LITERATURE AND DENTISTRY IN GENERAL, AND DR. ALLEN A LITTLE.

BY WM. A. PEASE.

THE lassitude and disinclination for labor and mental activity, arising from the heat of the summer, is past. The days are shortening, the evenings lengthening, and the hours in which the dentist is exempt from laboring at the chair are materially increased. With this exemption from active daily duty come other and equally as important duties, which should receive his serious attention and occupy that time, which, but for the shortened days, he would devote to his patients. Indeed, this additional labor is justly their due, as it is also due to his profession. It is due to them in the acquisition of a more extended and varied education, which will enlarge his sphere of influence and usefulness as a dentist and a man. It is due to his profession, not only as to the additional weight and importance he will thereby give it, but also, in furnishing it the record of his accruing experience and the result of his more mature deliberations. Thus, however much he may be inclined to ease, or whatever attractions society may throw around him, he should enjoy them but temperately, and reserve his best powers for his profession and the serious duties of life. It is, indeed, a self-denial, after the labors of the day; when the muscles are weary, the system relaxed, the nerves depressed, the blood is but half ærated, and the lungs are laboring heavily to throw off the impurities and the extra carbon of the system and that acquired from the breath of others, when he feels an instinctive desire, almost amounting to a necessity, to go out from his office; and casting care and thought away, to revel awhile in mere automatic existence; and when he feels that if he could but do it, and inhale the breath of Old Boreas, whose teeth are icicles, from whose huge lungs comes nothing putrescible or aught but purity; that every nerve would titillate and laugh to exhilaration; at such a time it is indeed a self-denial to sit down quietly in the office and compose himself for study or the toils of composition; and in the language of Virgil, though spoken of a very different place and different kind of labor, it can truly be said—hic opus—hoc labor est.

It is with emotions such as these that the writer of this relinquishes a walk in a beautiful moonlight evening, to discharge a duty to himself and his profession, which he feels has been very unnecessarily, wantonly, and perhaps maliciously thrust upon him, for no other purpose than to injure him and gain a selfish end. If, under these circumstances, during the course of this article, he should sometimes write with an earnestness and point that his cool and comfortable readers may think somewhat warm, he begs of them to recollect the moonlight, the cool and bracing air without and the gas light and stifling air within.

A professional man is a member of one of the learned professions. By reason of that membership he is supposed to have a knowledge of the learning of that profession, of the English language, if it is his native tongue, and also, to have a liberal or classical education. The acquisition of that knowledge is supposed, by virtue of its eliminating, elevating and ennobling properties, to have raised him above the standard of ordinary men; to have made him despise all meanness, trickery and pretense; to love truth and abhor falsehood or its semblance, and to have become a fit companion for learned and honorable men. As his education and associations are high, his conduct should be equally elevated, and free from all the tricks and petty devices that might be tolerated in members of a craft or trade; but which, on him, will leave an enduring and damaging stain. Educated to this high point, his sensibilities are acute and his perceptions of conduct clear and quick; he will accordingly dread as much any act, or the utterance of a word that could subject him to the curl of a lip, a just criticism, or a sarcasm, as a thief would the bastinado. If, rising above the ordinary duties of his profession, he essays to become a teacher, a professional censor, or to discharge the more difficult role of a reviewer, it is supposed he will bring to his vocation a more varied education, a more laborious and thoughtful preparation of his article, in order that he or his profession may suffer no detriment from his negligence or ignorance. Thus it will be perceived that the qualifications necessary for a professional writer or teacher are not light or easily attained; and that the responsibilities incident to the discharge of such vocation rest heavily upon the strongest shoulders. If such are the responsibilities of an ordinary member of a profession, how much greater are those of one who acts in a representative capacity as the first officer of a learned society or a professional convention, which must be honored or dishonored in the eyes of the world by his conduct, and from whom, at least, it has a right to expect, in his recorded utterances, words of weight and of wisdom!

In calling the attention of the profession, in a previous number of the Register, to a review of an article of mine, by Dr. Allen, I acted on the belief, founded on the internal evidence of the review, that the unusual manner of quotation and commenting on the article in review arose from ignorance and a mistaken idea of the duties of a reviewer, and not from ill will or a desire to misrepresent or injure me. His object was purely selfish. He wished to discredit me as a writer, because I had stated that dentistry, as a profession, was of recent origin. That the manufacturers of artificial dentures were mechanics, at least for the time being, and that single gum teeth on gold plate made the best practical artificial denture yet devised. His apparent objects in trying to discredit these statements were several. In addition to his other duties, he was largely engaged in the manufacture of artificial dentures, which made him unnecessarily sensitive on that point; and he held the patent right for the manufacture of that kind of artificial denture called continuous-gum, which he evidently thought would be in a very bad way until that article was discredited, if it had not already gone into a decline. Hence his first review, of which some of the errors and quotations have already been noticed and commented on; and hence the second, in which he has not only retained the objectionable manner of quotation of the first, but he has absolutely degenerated both in matter and style; and so faulty is his language and his grammar, that he seems to have forgotten that he was writing for English readers. It is difficult to conceive how a man of ordinary sensibility, after his attention had been called to his violation of the commonest decencies and rules of a reviewer, should subject himself to a severer criticism by continuing the same practice in a succeeding number. But such is the case in the present instance. It looks badly. It looks more as if it was done after a cool calculation, than from ignorance or inadvertence. As if he had diligently studied the article he was reviewing, and selected from it in different places, paragraphs, which, separated from the context and brought together, aided by adroitly interposing a sentence of his own here and there; that implied rather than declared, would best serve his purpose and lead the casual reader to suppose that the kind of artificial denture I had recommended for its superiority I had also condemned in the same article. Indeed, in a subsequent sentence he actually makes this declaration; unless there is a typographical error, which is not to be presumed. Even if this were so, what advantage it would be to Dr. Allen it is difficult to conceive, as it would only show an unbecoming negligence in writing, or a typographical error. But the reader will say, grant all this to be so—grant that, allowing his interest to overshadow his judgment, in his anxiety to write a telling review and make the best display possible, his conduct has been exceptionable—a little tricky, and that he has not been governed by those honorable rules that should govern professional men—grant all this, but of what interest is it to the profession? it is simply a contest between you and Dr. Allen, that in no way concerns us. Hold a minute! In this, my dear reader, you are a little too fast. You must recollect that the character and honor of all professions is the sum of that of their individual members; and that the deliberately published article of a presiding officer of one of its societies or conventions will be taken at home and abroad to represent the highest intelligence and manhood of the members thereof. Take that view of the case; such an article should not be allowed to go abroad without a protest; and neither should it be allowed to appear without a notice, lest it should be taken as a model by some young and inexperienced member. Hence this article.

Here it may be proper to add that no one can be more sensible of the deficiencies of the articles reviewed by Dr. Allen than the writer. The estimate there given of the comparative value of the different materials on which artificial teeth are inserted was written after much thought, and it was finally concluded that, however well individual members may succeed with other materials, taking the average skill of all the dentists (speaking in the popular sense and in deference to Dr. Allen) of the whole country into consideration, that single gum teeth on gold plate would be found the best, and to be the most satisfactory. This was only the expression of individual opinion. It was not calculated to give any one the fidgets, or to develop the *globus historicus*. And if Dr. Allen or any one else had chosen to state calmly and dispassionately his dissent from it, and to give his reasons therefor, it would have been kindly received as a review taken from a different stand-point, and if conclusive, endorsed. Turning, then, to the quotations of the review, let us see if the spirit which animated them or the ignorance there displayed, whichever it may be, is sufficient to warrant the serious charges here made, for if not, the writer has very much mistaken their tenor and import.

To my enumeration of the advantages and disadvantages of the different methods of inserting artificial teeth and the bases upon which they are made, the Doctor does not seem to take exception; for he says:

"It is not proposed to discuss the question in this article, but simply give his own statements with reference to this (best) mode of setting artificial teeth; for his alternate views present all that need be said pro and con."

Dr. Allen's first quotation is as follows:

"It is not surprising that a metal so beautiful as gold, so incorruptible and plastic, and one, withal, of so great intrinsic value, should be considered almost instinctively by the profession and the community as one eminently suitable to be a base for artificial teeth. Alloyed and adulterated as it often has been, until it was sometimes difficult to determine whether it predominated over a mixture of silver and copper; it has nevertheless, maintained its position in the professional and popular estimation. And it is believed that single gum-teeth well mounted upon good gold plate, make the best practical artificial denture that has yet been devised."

To this the Doctor replies: "*On the next page but one he spoils his golden picture by the following contra sentence.*" *Contra to what?* not surely to sets of teeth on gold, for these we recommend. But here is what he calls the contra sentence:

"A set of artificial teeth is a piece of mechanism—nothing more. It is made of light, frail and fracturable materials; and it is believed that the average duration of sets of artificial teeth will not exceed six years, while many of them are a constant source of trouble and expense from the first.'

To which the Doctor says: "*If this be true, it is certainly high time that a better method should be adopted.*" The italics are mine. A better method than what? Not surely than that on gold, for the paragraph did not refer to that. But it did refer to sets of teeth in general—to all kinds of artificial dentures, whether made on gold, silver, platina, or rubber. So that if a better method should be adopted, it must be something entirely new—undiscovered. But this was not what Dr. Allen intended. He wished to have it inferred that if a set of teeth on gold was so worthless, short-lived and troublesome, that those on continuous-gum would be just what is wanted. He would not have a new and better material brought before the profession to supersede all others, for his interests in continuous-gum are too great for that.

The manner of quoting the paragraph under consideration, which Dr. Allen calls *contra*, is so extraordinary, and it is such a violation of every rule of criticism, that it needs further notice, in order to show to what despicable means a stickler for professional honor can descend. The paragraph was taken from the middle of that part of the article in review, which relates to continuous-gum. It is a part of the subject; used in illustration of it; and designed to show that sets of teeth, called continuous-gum, for reasons given immediately before and after, are not as desirable as those mounted on gold. Hence it related to continuous-gum, if it related to any particular kind of artificial denture; and applied to it all of those undesirable qualities which Dr. Allen would have it give to sets of teeth on gold. And yet, Dr. Allen has had the effrontery and shamelessness to take this paragraph from the place where it belongs, and to which it relates, and to quote it in connection with another part of the article, describing the excellence and superiority of sets of teeth on gold, in order to disparage that kind of dentures, and nullify what had been said in favor of it; for he says: "*he spoils his golden picture by the following contra sentence.*" And then immediately after the quotation, says: "*If this be true, it is certainly high time that a better method should be adopted.*" Can impudence go further?

The two quotations, by Dr. Allen, I have just been considering are at the commencement of a long review. There are others of like character, having his peculiarities, and equally as obnoxious to criticism, which I had, at first, intended to notice; but I find the subject so disgusting and sickening, that I have not heart or stomach to proceed farther. I feel humiliated, as a dentist, that such reviews should have been written, and that they found utterance to the dentists of the world through the dental journals. They certainly are not creditable to the dental literature of America; their effect must be damaging, the more so, as they come from the first officer of a dental convention. With these remarks this article closes, so far as it relates to his quotations.

In the series of articles I have been writing, entitled, Who are Dentists? and which, if time and opportunity permit, I intend to conclude before the advent of another summer, I

called the manufacturers of artificial dentures mechanics. Dr. Allen has seized upon that term, and exhausted his ingenuity in trying to convert it into a disparagement of that kind of labor. This is no more than I might have expected from him ; it is fit that it should be done by so able a reviewer, as it rounds off his review to its fit and natural close. Hitherto, I had supposed that the word dentist was self-defining ; that it applied to that man who operates on the teeth, or treats them when diseased. Webster defines it as "One whose occupation is to clean and extract teeth, or repair them when diseased." A surgeon dentist is a man who, in addition to the ordinary labors of a dentist, performs surgical operations on the mouth and adjoining parts. A mechanical dentist, or dental mechanic, the terms are convertible, is a man who makes artificial dentures. Unlike the prefix surgeon, the term is valuable in showing what kind of a mechanic the man is. It specifies, determines, and defines. But having determined his position, it by no means follows that that determination carries with it any disparagement or degradation. It simply says that the skill he has, or the labor he performs, is not professional, or such as entitles him to membership in a learned profession, any more than he would be were he a manufacturer of a watch or spoon. The labor and vocation in itself is honorable, as all labor is ; and it is a matter of taste as to whether it is not as pleasant and desirable as that at the chair, it certainly is not as responsible. But to prove that I have not underrated the kind or quality of labor performed in the shop, or as Dr. Allen would probably term it, the laboratory, let us enter and see. The first we meet is a man having his sleeves rolled up, sooty and sweaty as any Vulcan, engaged at the fire melting and refining gold ; anon he is hammering it at the anvil like a smith ; now he is dabbling in moulding sand, like a child making mud cakes in the gutter ; again he is using his files and punches or puffing through his blow-pipe, and at last he is scorched to blear-eyedness, gazing into glowing muffles, watching the baking of continuous-gum. These are the representative labors of the shop. To say that they are professional is simply absurd—it shocks all common sense. That this is so, the common sense of mankind and the common use of language alike declare. In this connection, I am constrained to produce another authority, even at the risk that it will fall with such stunning weight upon the head of Dr. Allen as to produce concussion of the brain or other cerebral disturbance, and thus increase still more his comatose condition. Webster defines professional to be "The business one professes to understand and to follow for subsistence; calling; vocation; employment; as, the learned professions. We speak of the professions of a clergyman, of a lawyer, and of a physician or surgeon. But the word is not applied to any occupation merely mechanical."

In closing this article, I will only add that while there are too many dentists who are bad pluggers, and who exhibit but little knowledge of the diseases of the teeth and soft tissues, the most of the failures that I have seen, either in the East or West, come from mechanics, who can make a good set of teeth, so far as construction is concerned. These persons claim to be operators, and it is their operations that constitute the great bulk of the failures, which discourage the community and disgrace dentistry. They too often accelerate the loss of the teeth.

